# Crystal structure of bis­(3-bromo­mesit­yl)(quino­lin-1-ium-8-yl)boron(III) tribromide

**DOI:** 10.1107/S2056989015015467

**Published:** 2015-08-29

**Authors:** Jungho Son, Sem Raj Tamang, James D. Hoefelmeyer

**Affiliations:** a Department of Chemistry, University of South Dakota, 414 E. Clark St, Vermillion, SD 57069, USA

**Keywords:** crystal structure, frustrated Lewis pair, halogen, heterolysis, electrophilic aromatic substitution

## Abstract

The title compound, C_27_H_26.82_BBr_2.18_N^+^·Br_3_^−^, is a cationic tri­aryl­borane isolated as its tribromide salt. The aryl substituents include a protonated 8-quinolyl group and two 3-bromo­mesityl groups. The mol­ecule was prepared on combination of 3:1 Br_2_ and dimesit­yl(quinolin-8-yl)borane in hexa­nes. The refinement of the structure indicated a degree of ‘over-bromination’ (beyond two bromine atoms) for the cation. There are two tribromide ions in the asymmetric unit, both completed by crystallographic inversion symmetry.

## Abstract 2

The structure of bis­(3-bromo­mesit­yl)8-quinolyliniumboron(III) tribromide is reported: the refinement indicates that a degree of ‘over-bromination’ of the cation has occurred.

## Chemical context   

We recently prepared the preorganized unimolecular frustrated Lewis pair mol­ecule 8-quinolyldimesitylborane (Son *et al.*, 2010 ▸) and hypothesized that it could participate in the heterolytic cleavage of mol­ecular bromine. Halogen addition to a frustrated Lewis pair was recently reported in the literature (Frömel *et al.*, 2012 ▸). The combination of 8-quinolyldimesitylborane with three equivalents of Br_2_ in hexa­nes led to precipitation of the title compound. Features of the structure suggest heterolytic cleavage of Br_2_ occurred at the frustrated Lewis pair site. The bromination of the mesityl groups is likely due to electrophilic aromatic substitution from a brominium ion that yields HBr, manifest as a proton on the quinoline nitro­gen atom and bromide bound to mol­ecular bromine to form the tribromide ion. Alternatively, radical bromination of the solvent (hexa­ne) yields HBr; however, a radical mechanism is not likely for the bromination of mesityl groups. Typically bromination of aromatics is performed with a Lewis acid catalyst and occurs through an electrophilic aromatic substitution mechanism.
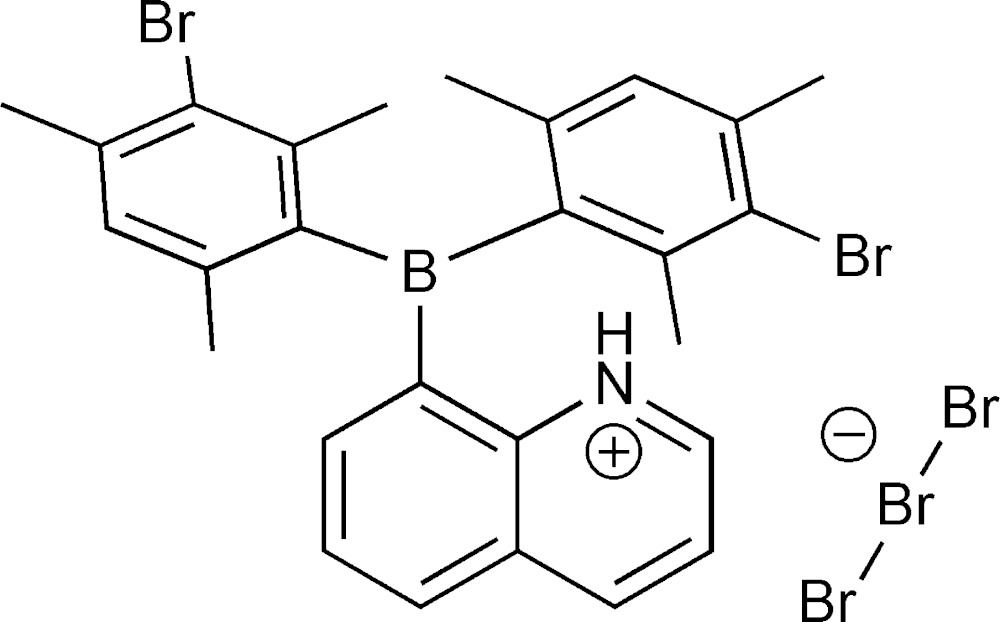


## Structural commentary   

**Figure 1:**
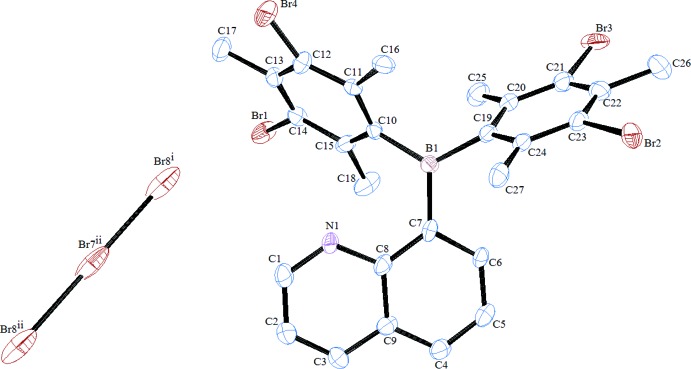
The mol­ecular structure of the title compound. Hydrogen atoms are omitted for clarity. Displacement ellipsoids are shown at the 30% probability level. [Symmetry codes: (i) 1 − *x*,1 − *y*,1 − *z*; (ii) 1 + *x*, *y*, *z*.]

The title compound crystallizes in the space group *P* 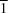, and contains one cation and two half tribromide ions (completed by inversion symmetry) in the asymmetric unit. The cation (Fig. 1 ▸) features a planar three-coordinate tri­aryl­borane with two 3-bromo­mesityl groups and an 8-quinolyl group. The nitro­gen atom is protonated and the positive charge is balanced by the presence of a tribromide anion, Br_3_^−^. The tribromide anions are shared between asymmetric units of the crystal, such that each unit contains two halves of an anion (Br5 and Br7 lie on crystallographic inversion centers). The Br5—Br6 distance is 2.5427 (11) Å and the Br7—Br8 distance is 2.546 (2) Å. Other bond distances and angles are given on Table 1 ▸. The mesityl groups are brominated at the *meta* positions such that one position is nearly completely brominated while the other *meta* position on the same ring is brominated to a much lesser extent. The best solution was found with refined bromine occupancy at the *meta* positions (C10 ring: Br1 = 0.95, Br4 = 0.09 for a total Br count of 1.04 on the ring; C19 ring: Br2 = 0.89, Br3 = 0.24 for a total Br count of 1.13 on the ring). The balance of electron density at the positions is accounted by partial hydrogen atoms at a reciprocal value of the bromine occupancy to give an overall formulation for the cation of C_27_H_26.82_BBr_2.18_N^+^.

## Supra­molecular features   

**Figure 2:**
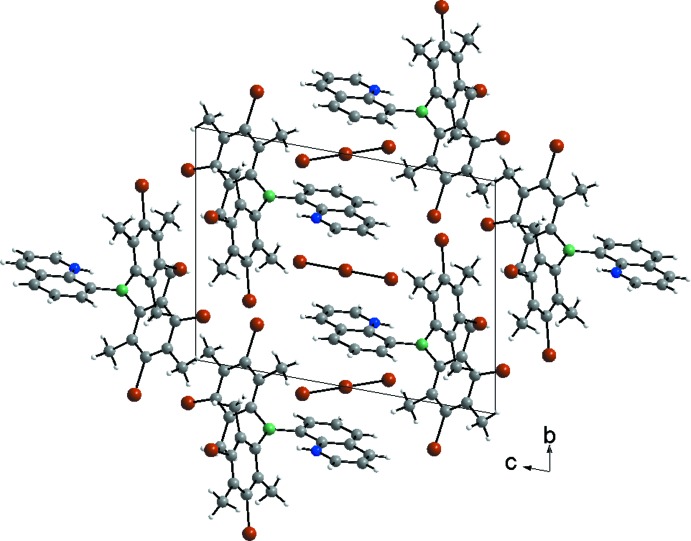
Packing diagram of bis­(3-bromo­mesit­yl)(quinolin-1-ium-8-yl)boron(III) tribromide in the crystal (C: gray, H: white, B: green, N: blue, Br: brown)

The cations are arranged in rows that propagate along the *a*-axis direction wherein each cation is in the same orientation due to translation along the row. Inversion centers are located on the dimesitylboryl side of the row, just beyond the brominated mesityl groups, and the packing of the cations in the crystal results in inter­digitated parallel quinolinium rings; these symmetrically sandwich a tribromide anion, such that the central atom of the anion is located at an inversion center. A packing diagram is shown in Fig. 2 ▸.

## Database survey   

A search in the Cambridge Structural Database (Groom & Allen, 2014 ▸) for structures with the tribromide anion revealed 162 hits while a search for structures with the dimesitylboryl fragment revealed 539 hits. Among these are several structures of planar organic aromatic cations as tribromide salts. There are examples that display a cationic aromatic ring–tribromide–cationic aromatic ring motif (Manna *et al.*, 2014 ▸), including 8-quinolinium derivatives (Müller *et al.*, 2010 ▸; Rybakov *et al.*, 2013 ▸) similar to the title compound. Alternatively, non-sandwich-type packing modes were found (Dean *et al.*, 2009 ▸) including structures that feature π-stacking between aromatic cations (Bakshi *et al.* (1996 ▸), even 8-quinolinium derivatives (Thone *et al.* (2010 ▸).

## Synthesis and crystallization   

Reactions were performed using Schlenk and glovebox techniques under an atmosphere of N_2_ using dried and distilled solvents. Dimesit­yl(8-quinol­yl)borane was prepared according to the literature (Son *et al.*, 2010 ▸). A round-bottom air-free flask was charged with 110 mg (0.29 mmol) dimesit­yl(8-quinol­yl)borane and 20 ml hexa­nes. In a separate flask, 2 ml of a solution of 5% Br_2_ in CCl_4_ (1 mmol Br_2_) was added to 10 ml hexa­nes and subjected to one freeze–pump–thaw cycle. The Br_2_ solution was transferred to the borane solution *via* a cannula at room temperature with stirring, and immediately a light-yellow precipitate formed. The solvent was removed *in vacuo*. Di­chloro­methane was added to the solid reside into which the title compound was dissolved; remaining insolubles were filtered off. Pale-yellow prisms of the title compound were grown by vapor diffusion of pentane into the methyl­ene chloride solution.

## Refinement   

Crystal data, data collection, and structure refinement details are summarized in Table 2 ▸. C-bound H atoms were refined using a riding model with C—H = 0.95 or 0.98 Å and with *U*_iso_(H) = 1.2 or 1.5*U*_eq_(C). The N-bound H atom was freely refined. 

## Supplementary Material

Crystal structure: contains datablock(s) I. DOI: 10.1107/S2056989015015467/hb7403sup1.cif

Structure factors: contains datablock(s) I. DOI: 10.1107/S2056989015015467/hb7403Isup2.hkl

CCDC reference: 1419502

Additional supporting information:  crystallographic information; 3D view; checkCIF report

## Figures and Tables

**Table 1:** Selected geometric parameters (, )

B1C7	1.579(14)	B1C19	1.588(14)
B1C10	1.598(13)	Br3C21	1.690(12)
Br1C14	1.901(9)	C23Br2	1.905(10)
			
C7B1C10	121.6(8)	C10B1C19	121.0(8)
C7B1C19	117.2(8)		

**Table 2:** Experimental details

Crystal data	
Chemical formula	C_27_H_26.82_BBr_2.18_N^+^Br_3_
*M*_r_	789.66
Crystal system, space group	Triclinic, *P* 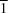
Temperature (K)	100
*a*, *b*, *c* ()	8.8469(10), 11.2365(13), 14.7528(18)
, , ()	79.600(2), 85.158(2), 87.994(2)
*V* (^3^)	1437.0(3)
*Z*	2
Radiation type	Mo *K*
(mm^1^)	7.25
Crystal size (mm)	0.44 0.22 0.14

Data collection	
Diffractometer	Bruker SMART CCD
Absorption correction	Multi-scan (*SADABS*; Bruker, 2008 ▸)
*T*_min_, *T*_max_	0.161, 0.362
No. of measured, independent and observed [*I* > 2(*I*)] reflections	14439, 5310, 3409
*R*_int_	0.052
(sin /)_max_ (^1^)	0.605

Refinement	
*R*[*F* ^2^ > 2(*F* ^2^)], *wR*(*F* ^2^), *S*	0.070, 0.156, 1.10
No. of reflections	5310
No. of parameters	341
H-atom treatment	H atoms treated by a mixture of independent and constrained refinement
_max_, _min_ (e ^3^)	1.19, 1.37

Computer programs: *SMART* and *SAINT* (Bruker, 2008 ▸), *SHELXTL* (Sheldrick, 2008 ▸).

## supplementary crystallographic information

### Crystal data

**Table:**

C_27_H_26.82_BBr_2.18_N^+^·Br_3_^−^	*Z* = 2
*M**_r_* = 789.66	*F*(000) = 764
Triclinic, *P*1	*D*_x_ = 1.825 Mg m^−^^3^
*a* = 8.8469 (10) Å	Mo *K*α radiation, λ = 0.71073 Å
*b* = 11.2365 (13) Å	Cell parameters from 4521 reflections
*c* = 14.7528 (18) Å	θ = 2.5–25.3°
α = 79.600 (2)°	µ = 7.25 mm^−^^1^
β = 85.158 (2)°	*T* = 100 K
γ = 87.994 (2)°	Prism, pale yellow
*V* = 1437.0 (3) Å^3^	0.44 × 0.22 × 0.14 mm

### Data collection

**Table:**

Bruker SMART CCD diffractometer	5310 independent reflections
Radiation source: sealed tube	3409 reflections with *I* > 2σ(*I*)
Graphite monochromator	*R*_int_ = 0.052
ω scans	θ_max_ = 25.5°, θ_min_ = 1.8°
Absorption correction: multi-scan (*SADABS*; Bruker, 2008)	*h* = −10→10
*T*_min_ = 0.161, *T*_max_ = 0.362	*k* = −13→13
14439 measured reflections	*l* = −17→17

### Refinement

**Table:**

Refinement on *F*^2^	0 restraints
Least-squares matrix: full	Hydrogen site location: mixed
*R*[*F*^2^ > 2σ(*F*^2^)] = 0.070	H atoms treated by a mixture of independent and constrained refinement
*wR*(*F*^2^) = 0.156	*w* = 1/[σ^2^(*F*_o_^2^) + (0.0279*P*)^2^ + 15.1288*P*] where *P* = (*F*_o_^2^ + 2*F*_c_^2^)/3
*S* = 1.10	(Δ/σ)_max_ < 0.001
5310 reflections	Δρ_max_ = 1.19 e Å^−^^3^
341 parameters	Δρ_min_ = −1.37 e Å^−^^3^

### Special details

**Table:**

Geometry. All e.s.d.'s (except the e.s.d. in the dihedral angle between two l.s. planes) are estimated using the full covariance matrix. The cell e.s.d.'s are taken into account individually in the estimation of e.s.d.'s in distances, angles and torsion angles; correlations between e.s.d.'s in cell parameters are only used when they are defined by crystal symmetry. An approximate (isotropic) treatment of cell e.s.d.'s is used for estimating e.s.d.'s involving l.s. planes.

### Fractional atomic coordinates and isotropic or equivalent isotropic displacement parameters (Å^2^)

**Table:**

	*x*	*y*	*z*	*U*_iso_*/*U*_eq_	Occ. (<1)
C1	0.5396 (12)	0.6737 (8)	0.5329 (8)	0.047 (3)	
H1	0.6369	0.6441	0.5502	0.057*	
C2	0.5090 (13)	0.6857 (10)	0.4432 (8)	0.055 (3)	
H2	0.5840	0.6666	0.3978	0.066*	
C3	0.3669 (13)	0.7260 (10)	0.4196 (8)	0.054 (3)	
H3	0.3434	0.7332	0.3572	0.065*	
C4	0.1112 (12)	0.8030 (8)	0.4622 (7)	0.047 (3)	
H4	0.0848	0.8145	0.4001	0.056*	
C5	0.0096 (12)	0.8311 (8)	0.5300 (7)	0.045 (3)	
H5	−0.0890	0.8597	0.5147	0.054*	
C6	0.0463 (11)	0.8191 (8)	0.6208 (7)	0.036 (2)	
H6	−0.0270	0.8424	0.6656	0.043*	
C7	0.1880 (10)	0.7735 (8)	0.6488 (7)	0.036 (2)	
C8	0.2927 (11)	0.7434 (8)	0.5796 (7)	0.039 (2)	
C9	0.2561 (12)	0.7568 (8)	0.4849 (7)	0.040 (2)	
C10	0.3368 (10)	0.6643 (8)	0.8014 (6)	0.032 (2)	
C11	0.4517 (11)	0.7001 (8)	0.8499 (6)	0.035 (2)	
C12	0.5639 (11)	0.6170 (10)	0.8818 (7)	0.046 (3)	
H12_b	0.6450	0.6451	0.9101	0.055*	0.911 (5)
C13	0.5647 (11)	0.4975 (9)	0.8725 (7)	0.040 (2)	
C14	0.4479 (12)	0.4607 (8)	0.8295 (6)	0.039 (2)	
H14_c	0.4436	0.3778	0.8244	0.046*	0.047 (5)
C15	0.3328 (11)	0.5414 (9)	0.7922 (7)	0.042 (2)	
C16	0.4624 (12)	0.8290 (9)	0.8694 (7)	0.043 (3)	
H16A	0.3987	0.8841	0.8287	0.065*	
H16B	0.5680	0.8549	0.8579	0.065*	
H16C	0.4273	0.8304	0.9340	0.065*	
C17	0.6889 (12)	0.4110 (10)	0.9100 (8)	0.058 (3)	
H17A	0.6436	0.3361	0.9444	0.087*	
H17B	0.7458	0.4484	0.9513	0.087*	
H17C	0.7577	0.3923	0.8586	0.087*	
C18	0.2075 (14)	0.4930 (9)	0.7468 (8)	0.061 (3)	
H18A	0.1540	0.4300	0.7914	0.092*	
H18B	0.2510	0.4587	0.6937	0.092*	
H18C	0.1361	0.5589	0.7260	0.092*	
C19	0.1088 (11)	0.8376 (8)	0.8148 (7)	0.037 (2)	
C20	0.0109 (12)	0.7798 (9)	0.8880 (7)	0.044 (3)	
C21	−0.0971 (12)	0.8503 (11)	0.9318 (7)	0.051 (3)	
H21_a	−0.1628	0.8109	0.9816	0.061*	0.761 (5)
C22	−0.1111 (12)	0.9745 (11)	0.9049 (7)	0.049 (3)	
C23	−0.0117 (12)	1.0276 (9)	0.8338 (7)	0.044 (3)	
H23_d	−0.0193	1.1128	0.8142	0.052*	0.106 (5)
C24	0.1006 (11)	0.9639 (9)	0.7882 (7)	0.039 (2)	
C25	0.0069 (13)	0.6444 (10)	0.9180 (8)	0.059 (3)	
H25A	−0.0305	0.6078	0.8689	0.088*	
H25B	−0.0609	0.6244	0.9744	0.088*	
H25C	0.1093	0.6130	0.9301	0.088*	
C26	−0.2310 (14)	1.0438 (13)	0.9548 (9)	0.075 (4)	
H26A	−0.3066	1.0785	0.9121	0.113*	
H26B	−0.1833	1.1090	0.9775	0.113*	
H26C	−0.2807	0.9888	1.0071	0.113*	
C27	0.2097 (12)	1.0305 (9)	0.7132 (7)	0.047 (3)	
H27A	0.1528	1.0739	0.6627	0.070*	
H27B	0.2808	0.9724	0.6896	0.070*	
H27C	0.2661	1.0886	0.7388	0.070*	
B1	0.2151 (13)	0.7594 (9)	0.7547 (7)	0.033 (3)	
N1	0.4375 (10)	0.7022 (7)	0.5977 (6)	0.039 (2)	
Br1_c	0.43924 (16)	0.29439 (10)	0.82058 (9)	0.0618 (6)	0.953 (5)
Br2_d	−0.03034 (16)	1.19837 (10)	0.79562 (9)	0.0541 (5)	0.894 (5)
Br3_a	−0.2186 (6)	0.8077 (6)	1.0259 (3)	0.070 (3)	0.239 (5)
Br4_b	0.6985 (17)	0.619 (2)	0.9386 (13)	0.112 (11)	0.089 (5)
H1N	0.474 (18)	0.700 (14)	0.655 (11)	0.134*	
Br5	0.5000	0.0000	0.5000	0.0421 (4)	
Br6	0.35660 (13)	0.06362 (10)	0.35619 (8)	0.0532 (4)	
Br7	0.0000	0.5000	0.5000	0.0837 (8)	
Br8	0.15880 (17)	0.49848 (12)	0.34799 (12)	0.0921 (6)	

### Atomic displacement parameters (Å^2^)

**Table:**

	*U*^11^	*U*^22^	*U*^33^	*U*^12^	*U*^13^	*U*^23^
C1	0.051 (7)	0.035 (6)	0.058 (7)	0.015 (5)	−0.007 (6)	−0.016 (5)
C2	0.059 (8)	0.059 (7)	0.046 (7)	0.020 (6)	−0.002 (6)	−0.013 (6)
C3	0.060 (8)	0.059 (7)	0.047 (7)	0.017 (6)	−0.006 (6)	−0.026 (6)
C4	0.063 (8)	0.035 (6)	0.047 (7)	0.013 (5)	−0.020 (6)	−0.014 (5)
C5	0.043 (6)	0.033 (5)	0.060 (7)	0.005 (5)	−0.018 (6)	−0.006 (5)
C6	0.035 (6)	0.028 (5)	0.042 (6)	0.010 (4)	−0.007 (5)	−0.002 (4)
C7	0.029 (5)	0.026 (5)	0.052 (6)	0.006 (4)	−0.007 (5)	−0.003 (4)
C8	0.043 (6)	0.026 (5)	0.049 (6)	0.009 (4)	−0.015 (5)	−0.008 (4)
C9	0.053 (7)	0.029 (5)	0.042 (6)	0.005 (5)	−0.017 (5)	−0.014 (4)
C10	0.036 (6)	0.028 (5)	0.030 (5)	0.006 (4)	0.001 (4)	−0.005 (4)
C11	0.038 (6)	0.044 (6)	0.023 (5)	0.004 (5)	0.003 (4)	−0.004 (4)
C12	0.028 (6)	0.057 (7)	0.053 (7)	0.001 (5)	−0.008 (5)	−0.011 (5)
C13	0.033 (6)	0.048 (6)	0.038 (6)	0.006 (5)	0.002 (5)	−0.006 (5)
C14	0.056 (7)	0.022 (5)	0.036 (6)	0.005 (4)	0.000 (5)	−0.004 (4)
C15	0.045 (6)	0.047 (6)	0.035 (6)	0.003 (5)	−0.014 (5)	−0.003 (5)
C16	0.047 (6)	0.052 (6)	0.034 (6)	−0.004 (5)	−0.005 (5)	−0.015 (5)
C17	0.045 (7)	0.059 (7)	0.072 (8)	0.017 (6)	−0.016 (6)	−0.014 (6)
C18	0.082 (9)	0.027 (5)	0.079 (9)	0.001 (6)	−0.039 (7)	−0.005 (5)
C19	0.040 (6)	0.034 (5)	0.039 (6)	0.008 (4)	−0.016 (5)	−0.009 (4)
C20	0.046 (6)	0.053 (7)	0.034 (6)	0.014 (5)	−0.011 (5)	−0.006 (5)
C21	0.040 (6)	0.072 (8)	0.037 (6)	0.009 (6)	−0.002 (5)	0.000 (6)
C22	0.036 (6)	0.074 (8)	0.041 (6)	−0.001 (6)	−0.006 (5)	−0.017 (6)
C23	0.053 (7)	0.034 (5)	0.048 (6)	0.014 (5)	−0.023 (6)	−0.015 (5)
C24	0.038 (6)	0.043 (6)	0.040 (6)	0.009 (5)	−0.016 (5)	−0.012 (5)
C25	0.049 (7)	0.065 (8)	0.056 (7)	0.002 (6)	−0.003 (6)	0.007 (6)
C26	0.064 (9)	0.107 (11)	0.059 (8)	0.021 (8)	0.001 (7)	−0.037 (8)
C27	0.048 (7)	0.034 (6)	0.057 (7)	0.010 (5)	−0.010 (5)	−0.005 (5)
B1	0.040 (7)	0.028 (6)	0.029 (6)	0.003 (5)	−0.001 (5)	−0.001 (5)
N1	0.044 (5)	0.030 (4)	0.044 (5)	0.015 (4)	−0.008 (4)	−0.010 (4)
Br1_c	0.0883 (11)	0.0296 (7)	0.0724 (10)	0.0149 (6)	−0.0404 (8)	−0.0099 (6)
Br2_d	0.0710 (10)	0.0353 (7)	0.0590 (9)	0.0164 (6)	−0.0068 (7)	−0.0189 (6)
Br3_a	0.043 (3)	0.138 (6)	0.033 (3)	−0.012 (3)	−0.008 (2)	−0.021 (3)
Br4_b	0.037 (10)	0.22 (3)	0.085 (14)	0.036 (11)	−0.026 (8)	−0.052 (14)
Br5	0.0373 (8)	0.0411 (8)	0.0431 (9)	0.0049 (6)	0.0028 (7)	0.0012 (6)
Br6	0.0453 (7)	0.0616 (7)	0.0474 (7)	0.0047 (5)	−0.0052 (5)	0.0040 (5)
Br7	0.0685 (12)	0.0449 (10)	0.1295 (18)	−0.0202 (9)	−0.0623 (12)	0.0350 (10)
Br8	0.0755 (10)	0.0663 (9)	0.1269 (13)	−0.0174 (7)	−0.0613 (9)	0.0309 (8)

### Geometric parameters (Å, º)

**Table:**

C1—N1	1.333 (13)	C16—H16C	0.9800
C1—C2	1.355 (14)	C17—H17A	0.9800
C1—H1	0.9500	C17—H17B	0.9800
C2—C3	1.373 (14)	C17—H17C	0.9800
C2—H2	0.9500	C18—H18A	0.9800
C3—C9	1.395 (14)	C18—H18B	0.9800
C3—H3	0.9500	C18—H18C	0.9800
C4—C5	1.359 (14)	C19—C20	1.403 (14)
C4—C9	1.410 (13)	C19—C24	1.403 (13)
C4—H4	0.9500	B1—C19	1.588 (14)
C5—C6	1.388 (13)	C20—C21	1.409 (14)
C5—H5	0.9500	C20—C25	1.506 (14)
C6—C7	1.404 (12)	C21—C22	1.385 (15)
C6—H6	0.9500	Br3_a—C21	1.690 (12)
C7—C8	1.400 (13)	C21—H21_a	0.9500
B1—C7	1.579 (14)	C22—C23	1.373 (15)
C8—N1	1.378 (12)	C22—C26	1.513 (15)
C8—C9	1.440 (13)	C23—C24	1.402 (13)
C10—C11	1.402 (13)	C23—Br2_d	1.905 (10)
C10—C15	1.414 (13)	C23—H23_d	0.9500
B1—C10	1.598 (13)	C24—C27	1.512 (14)
C11—C12	1.390 (13)	C25—H25A	0.9800
C11—C16	1.534 (13)	C25—H25B	0.9800
C12—C13	1.374 (14)	C25—H25C	0.9800
C12—Br4_b	1.516 (17)	C26—H26A	0.9800
C12—H12_b	0.9500	C26—H26B	0.9800
C13—C14	1.370 (14)	C26—H26C	0.9800
C13—C17	1.514 (13)	C27—H27A	0.9800
C13—Br4_b	2.24 (2)	C27—H27B	0.9800
C14—C15	1.418 (13)	C27—H27C	0.9800
Br1_c—C14	1.901 (9)	N1—H1N	0.92 (15)
C14—H14_c	0.9500	Br5—Br6^i^	2.5427 (11)
C15—C18	1.507 (14)	Br5—Br6	2.5427 (11)
C16—H16A	0.9800	Br7—Br8	2.546 (2)
C16—H16B	0.9800	Br7—Br8^ii^	2.546 (2)
			
N1—C1—C2	122.1 (10)	H17A—C17—H17C	109.5
N1—C1—H1	119.0	H17B—C17—H17C	109.5
C2—C1—H1	119.0	C15—C18—H18A	109.5
C1—C2—C3	118.4 (10)	C15—C18—H18B	109.5
C1—C2—H2	120.8	H18A—C18—H18B	109.5
C3—C2—H2	120.8	C15—C18—H18C	109.5
C2—C3—C9	121.7 (10)	H18A—C18—H18C	109.5
C2—C3—H3	119.2	H18B—C18—H18C	109.5
C9—C3—H3	119.2	C20—C19—C24	119.8 (9)
C5—C4—C9	119.5 (9)	C20—C19—B1	119.9 (8)
C5—C4—H4	120.3	C24—C19—B1	119.8 (9)
C9—C4—H4	120.3	C19—C20—C21	118.8 (9)
C4—C5—C6	121.7 (9)	C19—C20—C25	123.3 (9)
C4—C5—H5	119.2	C21—C20—C25	117.7 (10)
C6—C5—H5	119.2	C22—C21—C20	122.5 (10)
C5—C6—C7	122.2 (9)	C22—C21—Br3_a	108.2 (9)
C5—C6—H6	118.9	C20—C21—Br3_a	129.2 (9)
C7—C6—H6	118.9	C22—C21—H21_a	118.9
C8—C7—C6	116.4 (9)	C20—C21—H21_a	118.6
C8—C7—B1	125.8 (8)	Br3_a—C21—H21_a	11.3
C6—C7—B1	117.8 (9)	C23—C22—C21	116.7 (10)
N1—C8—C7	121.9 (9)	C23—C22—C26	123.9 (11)
N1—C8—C9	116.4 (9)	C21—C22—C26	119.4 (11)
C7—C8—C9	121.7 (9)	C22—C23—C24	124.0 (9)
C3—C9—C4	123.1 (9)	C22—C23—Br2_d	117.2 (8)
C3—C9—C8	118.4 (9)	C24—C23—Br2_d	118.8 (8)
C4—C9—C8	118.5 (9)	C22—C23—H23_d	118.2
C11—C10—C15	118.3 (8)	C24—C23—H23_d	117.8
C11—C10—B1	121.5 (8)	Br2_d—C23—H23_d	1.0
C15—C10—B1	120.1 (8)	C23—C24—C19	118.0 (10)
C12—C11—C10	119.7 (9)	C23—C24—C27	120.4 (9)
C12—C11—C16	117.2 (9)	C19—C24—C27	121.6 (9)
C10—C11—C16	123.1 (8)	C20—C25—H25A	109.5
C13—C12—C11	123.3 (10)	C20—C25—H25B	109.5
C13—C12—Br4_b	101.3 (12)	H25A—C25—H25B	109.5
C11—C12—Br4_b	135.2 (13)	C20—C25—H25C	109.5
C13—C12—H12_b	118.7	H25A—C25—H25C	109.5
C11—C12—H12_b	118.1	H25B—C25—H25C	109.5
Br4_b—C12—H12_b	18.4	C22—C26—H26A	109.5
C14—C13—C12	117.2 (9)	C22—C26—H26B	109.5
C14—C13—C17	122.1 (9)	H26A—C26—H26B	109.5
C12—C13—C17	120.8 (9)	C22—C26—H26C	109.5
C14—C13—Br4_b	158.5 (9)	H26A—C26—H26C	109.5
C12—C13—Br4_b	41.7 (7)	H26B—C26—H26C	109.5
C17—C13—Br4_b	79.2 (8)	C24—C27—H27A	109.5
C13—C14—C15	122.6 (9)	C24—C27—H27B	109.5
C13—C14—Br1_c	118.5 (7)	H27A—C27—H27B	109.5
C15—C14—Br1_c	118.8 (8)	C24—C27—H27C	109.5
C13—C14—H14_c	118.8	H27A—C27—H27C	109.5
C15—C14—H14_c	118.5	H27B—C27—H27C	109.5
Br1_c—C14—H14_c	0.6	C7—B1—C10	121.6 (8)
C10—C15—C14	118.8 (9)	C7—B1—C19	117.2 (8)
C10—C15—C18	122.0 (8)	C10—B1—C19	121.0 (8)
C14—C15—C18	119.2 (9)	C1—N1—C8	123.0 (9)
C11—C16—H16A	109.5	C1—N1—H1N	115 (10)
C11—C16—H16B	109.5	C8—N1—H1N	122 (10)
H16A—C16—H16B	109.5	C14—Br1_c—H14_c	0.6
C11—C16—H16C	109.5	C23—Br2_d—H23_d	1.0
H16A—C16—H16C	109.5	C21—Br3_a—H21_a	13.8
H16B—C16—H16C	109.5	C12—Br4_b—C13	37.1 (7)
C13—C17—H17A	109.5	C12—Br4_b—H12_b	26.0
C13—C17—H17B	109.5	C13—Br4_b—H12_b	62.2
H17A—C17—H17B	109.5	Br6^i^—Br5—Br6	180.0
C13—C17—H17C	109.5	Br8—Br7—Br8^ii^	180.0

Symmetry codes: (i) −*x*+1, −*y*, −*z*+1; (ii) −*x*, −*y*+1, −*z*+1.
